# Mild Therapeutic Hypothermia Alters Hemostasis in ST Elevation Myocardial Infarction Patients

**DOI:** 10.3389/fcvm.2021.707367

**Published:** 2021-07-06

**Authors:** Thomas Scherz, Thomas M. Hofbauer, Anna S. Ondracek, Daniel Simon, Fritz Sterz, Christoph Testori, Irene M. Lang, Andreas Mangold

**Affiliations:** ^1^Division of Cardiology, Department of Internal Medicine II, Vienna General Hospital, Medical University of Vienna, Vienna, Austria; ^2^Department of Dermatology, Landesklinikum Wiener Neustadt, Wiener Neustadt, Austria; ^3^Department of Internal Medicine I, Cardiology, Klinikum Bamberg, Bamberg, Germany; ^4^Department of Emergency Medicine, Vienna General Hospital, Medical University of Vienna, Vienna, Austria; ^5^Department of Internal Medicine, Cardiology and Nephrology, Landesklinikum Wiener Neustadt, Wiener Neustadt, Austria

**Keywords:** mild therapeutic hypothermia, myocardial infarction, STEMI, hemostasis, platelets

## Abstract

**Background and Rationale:** Mild therapeutic hypothermia (MTH) is a concept to reduce infarct size and improve outcome after ST-segment elevation myocardial infarction (STEMI). In the STATIM trial, we investigated MTH as an additional therapy for STEMI patients. In the intention-to-treat set, 101 patients were included. No difference in primary and secondary endpoints measured by cardiac magnetic resonance imaging was found. Platelet activation and plasmatic coagulation are key in the pathophysiology of STEMI. In the present study, we investigated the effect of MTH on primary and secondary hemostasis in STEMI patients.

**Methods and Results:** Platelet function and morphology were assessed by routine blood count, aggregometry testing, and flow cytometry. Soluble platelet markers were determined by enzyme-linked immunosorbent assay (ELISA) testing. Plasmatic coagulation was measured throughout the study. Platelet count remained unchanged, irrespective of treatment, whereas platelet size decreased in both patient groups. Platelet aggregometry indicated increased platelet reactivity in the MTH group. Furthermore, higher adenosine diphosphate (ADP) plasma levels were found in MTH patients. Expression of glycoprotein IIb/IIIa was increased on platelets of STEMI patients treated with MTH. Lower patient temperatures correlated with longer clotting times and resulted in reduced pH. Lower pH values were positively correlated with longer clotting times.

**Conclusion:** Present data indicate longer clotting times and higher platelet reactivity in STEMI patients treated with MTH. These changes did not correspond to clinical bleeding events or larger infarct size.

## Introduction

Acute coronary syndrome (ACS) is amongst the leading causes of death worldwide (1). Infarct size is one of the best predictors of long-term outcome in ST-elevation myocardial infarction (STEMI) patients (2). While hyperthermia is associated with an unfavorable outcome (3), mild therapeutic hypothermia (MTH) has been studied in STEMI patients as adjunct to primary percutaneous coronary intervention (pPCI) to reduce final infarct size. Several studies tested MTH in various other clinical settings such as stroke (4), neonatal hypoxic encephalopathy (5) and myocardial infarction (6–9). In clinical routine, MTH is primarily used in patients suffering from cardiac arrest (10). However, a large trial did not indicate a mortality benefit for MTH in cardiac arrest patients (11). A meta-analysis including six randomized controlled trials showed a significant reduction of infarct size in myocardial infarction patients with anterior wall involvement by MTH. However, no effect on mortality or infarct size in the overall patient cohort could be observed (12). In the STATIM trial, 120 patients were included, of whom 101 remained in the final analysis. We did not observe any difference in primary and secondary endpoints by cardiac magnetic resonance (CMR) imaging 2 ± 4 and 195 ± 15 days after pPCI (13). Platelet activation, atherothrombosis and fibrin deposition are key mechanisms in STEMI, not only causing coronary artery occlusion but also microvascular obstruction, which leads to extended myocardial damage and unfavorable outcome (14). The impact of MTH on human platelet function and coagulation is limited. Differing results were reported on the effect of MTH on coagulation parameters in cardiac arrest patients (15, 16), and platelet reactivity was not changed by MTH in patients with cardiogenic shock (17). In the present prospective randomized controlled trial, we investigated the effect of MTH on primary and secondary hemostasis in STEMI patients.

## Materials and Methods

### Patients and Study Design

The present study is a substudy of the randomized, controlled, open-label, end-point blinded STATIM trial (13). One hundred twenty STEMI patients were allocated to MTH or normothermia. Randomization was conducted by an emergency physician in an out-of-hospital setting. Study patients were assigned 1:1 to target temperature management or normothermia. One hundred one patients remained in the intention-to-treat data set (54 normothermia group, 47 MTH group). Inclusion criteria were: (1) Age between 18 and 75, (2) at least 30 min of continuous typical chest pain, (3) anterior or inferior STEMI with ST-segment elevation of >0.2 mV in two contiguous leads, (4) duration of symptoms <6 h prior to first medical contact (FMC), and (5) immediate access to the catheterization laboratory. Exclusion criteria were: Cardiac arrest, a tympanic temperature below 35°C at presentation, history of myocardial infarction or PCI/coronary artery bypass grafting, chronic (New York Heart Association II–IV) or acute heart failure (Killip class II–IV), thrombolysis, infection, end-stage kidney disease, hepatic failure, recent stroke, hematological dyscrasias, oral anticoagulant treatment, severe pulmonary disease, known allergy to medications used in the trial, and being female of childbearing age (13). The MTH group received MTH treatment by surface cooling (EMCOOLS Flex.Pad^®^, EMCOOLS Emergency Medical Cooling Systems AG) and administration of ice cold saline (10 ml/kg for anterior STEMI; 20 ml/kg for inferior STEMI) at FMC. After arrival in the catheterization laboratory, an endovascular cooling catheter (Accutrol 14 Fr catheter and InnerCool RTx endovascular console, ZOLL Medical Corporation) was placed. Target temperature was set to 34 °C and maintained for 60 min after reperfusion. For shivering control, buspirone and meperidine were administered. Bleeding events were monitored for the first 45 days after study inclusion and classified according to the TIMI bleeding criteria (18).

### Routine Blood Tests

Blood draws were obtained at predetermined time points (FMC, pPCI, and 72 h after pPCI). Arterial blood was collected for blood gas analysis during pPCI. Patients did not receive any emergency medication prior to the FMC blood draws. Blood was immediately anticoagulated with EDTA (ethylenediaminetetraacetic acid), citrate or heparin, and transferred to the Clinical Department of Laboratory Medicine (General Hospital Vienna). Dilution effects were considered by normalizing consecutive blood draws to hematocrit from FMC. Extrinsic and common pathway of blood coagulation was tested by measuring prothrombin time (prothrombin time, given as percentage). Partial thromboplastin time (given as seconds) was used for testing the intrinsic and common pathway. All pH values shown in this study were measured at time of reperfusion.

### Platelet Aggregometry

Platelet function was measured by aggregometry (Multiplate^®^ Analyzer, Roche Diagnostics). Heparin anticoagulated blood was obtained for platelet aggregation measurements at FMC and at pPCI. For stimulation, arachidonic acid (ASPI-test), adenosine diphosphate (ADP, ADP-test) or thrombin receptor activating peptide-6 (TRAP-6, TRAP-test) were used, and diluted according to manufacturer's protocol (ASPI-test, ADP-test, TRAP-test; Roche Diagnostics).

### Flow Cytometry

Blood samples were immediately transferred to the laboratory facilities and incubated with the following fluorochrome-labeled antibodies at room temperature for 30 min: PAC-1 (glycoprotein IIb/IIIa) (BD Biosciences Pharmingen), CD45, CD14, CD16, glycoprotein Ib (CD42b), P-selectin (CD62p), PECAM-1 (CD31) and integrin alpha-V (CD51) (Biolegend). Erythrocytes were lysed by addition of BD FACS lysing solution (Becton Dickinson). Samples were analyzed using a 6-color BD Canto II (Becton Dickinson). Forward and side scatter were used to divide cell debris/platelets from viable cells. Platelets were identified by forward and sideward scatter as well as glycoprotein Ib (CD42b) positivity. Monocyte-platelet-aggregates (MPA) were defined by co-aggregation of monocytes and platelets. CD45 and CD14 were used for monocyte identification as previously described (19).

### Plasma Measurements

ADP plasma levels were evaluated by an ADP Assay Kit (Abcam). sP-Selectin (soluble P-selectin) was determined utilizing a Human sP-selectin Platinum ELISA (Thermo Scientific). For the quantification of thrombospondin (TSP-1) a Human Thrombospondin-1 Quantikine ELISA Kit (R&D Systems) was used. Soluble CD40L concentration was determined using a Human sCD40L (soluble CD40 ligand) Platinum ELISA Kit (Thermo Scientific). All assays were performed according to the manufacturers' protocol. All plates were measured by a VersaMax microplate reader (Molecular Devices). ADP Assay kit plates were read on a Varioskan Flash microplate reader (Thermo Scientific).

### Cardiac Magnetic Resonance Imaging

A 1.5 T system cardiac magnetic resonance (CMR) (Avanto Fit, Siemens Medical Systems) was performed for each patient 4 ± 2 days after pPCI to measure left ventricular function, infarct size, microvascular obstruction and myocardial salvage index. A second CMR was performed 195 ± 15 days after PCI. The detailed protocol is described elsewhere (13, 20).

### Statistics

Results are presented as mean ± standard deviation (SD) or median [interquartile range, IQR]. For normally distributed data, paired Students *t*-test was applied; otherwise, Wilcoxon signed rank test was used. The comparison between two groups was performed by a two-tailed unpaired *t*-test for normally distributed data, otherwise Mann-Whitney-*U*-test was employed. We used Δ values to compare value change over time between normothermia and MTH groups. Δ values of tested parameters were calculated by subtracting FMC values from pPCI values, followed by a two-tailed unpaired *t*-test or Mann-Whitney-*U*-test. Kolmogorov-Smirnov test and visual assessment of histograms were applied to test for normal distribution. An ANOVA with repeated measures was used to compare more than two groups. For multiple testing, Bonferroni-Holm correction was employed. Pearson correlation (r) was used for normally distributed data, otherwise data were calculated by Spearman rank correlation (r_s_). Chi-square test for independence was used to calculate a relationship between two categorical variables. A *p* < 0.05 was considered statistically significant. Statistical analyses were performed using IBM SPSS Statistics 26.0 (IBM). Figures were generated using GraphPad Prism 9.0 (GraphPad).

## Results

### Patient Characteristics and Outcome Parameters

In the STATIM trial, 120 patients were recruited between 2013 and 2016. 19 study patients were excluded after randomization. Causes for exclusion were: STEMI criteria not fulfilled (8 patients), Killip Class >I (1 patient), no availability of the cath lab (1 patient), history of myocardial infarction (2 patients), symptom onset >6 h (2 patients) and not suitable for CMR (3 patients). The intention-to-treat set consisted of 101 patients, of whom 47 patients were assigned to MTH treatment. A detailed flow chart of the STATIM trial is published in (13). Previous medication of patients including angiotensin converting enzyme inhibitor (ACE-I), acetylsalicylic acid (ASA), angiotensin receptor blocker (ARB), and statin intake is listed in Table 1. Statin treatment was significantly more prevalent in patients of the normothermia group (normothermia: *n* = 12, MTH: *n* = 3; *p* < 0.05; Table 1). Regular intake of any other medication including antidiabetic drugs (12 patients), vitamin D supplements (5 patients), non-steroidal anti-inflammatory drugs (NSAID) without ASA (4 patients), antidepressant drugs (6 patients), proton pump inhibitor (9 patients), amiodarone (1 patient), thyroid replacement medication (2 patients), alpha blockers (4 patients) was overall very low and showed no group differences. There was no correlation between any pre-existing medication of patients and tested parameters in this study (data not shown). All patients received acetylsalicylic acid (ASA), heparin and prasugrel or ticagrelor by the emergency physician prior to pPCI. Patients in the MTH group had a significantly lower temperature at reperfusion (normothermia: 36.3 ± 0.6°C, MTH: 34.4 ± 0.6°C; *p* < 0.0001; Table 1). There was no difference in the number of bleeding events. Detailed patient characteristics are displayed in Table 1. Measurements of routine laboratory testing did not differ between both groups at the time of reperfusion as illustrated in Table 2. An extended list of laboratory parameters is published elsewhere (13).

**Table 1:** Baseline characteristics and outcome parameters of patients in the STATIM trial.

	**Normothermia (*n* = 54)**	**MTH (*n* = 47)**	***p*-value**
Age, years	55 ± 12	58 ± 10	
Female sex, *n* (%)	10 (19)	10 (21)	
Hypertension, *n* (%)	25 (46)	13 (28)	
Diabetes, *n* (%)	10 (19)	5 (11)	
Dyslipidaemia, *n* (%)	17 (32)	10 (21)	
Current smoker, *n* (%)	30 (56)	26 (55)	
Family history of CAD, *n* (%)	12 (22)	12 (25)	
BMI >25 kg/m^2^, *n* (%)	43 (81)	28 (60)	<0.05
Previous medication			
ASA, *n* (%)	6 (11)	1 (2)	
Beta blocker, *n* (%)	7 (13)	4(9)	
ACE-I, *n* (%)	4 (7)	6 (13)	
ARB, *n* (%)	4 (7)	4 (9)	
Statin, *n* (%)	12 (22)	3 (6)	<0.05
Symptom onset to reperfusion, min	180 ± 87	192 ± 67	
Temperature at reperfusion, °C	36.3 ± 0.6	34.4 ± 0.6	<0.0001
Anterior wall infarction, *n* (%)	25 (46)	27 (57)	
Infarct size, ml	29 ± 23	27 ± 21	
Microvascular obstruction, ml	3 ± 5.8	2.2 ± 4	
Major bleedings, *n* (%)	0 (0)	1 (2)	
Death, *n* (%)	2 (4)	1 (2)	

*Results are displayed as mean ± standard deviation or absolute number (percent). Mann-Whitney-U-test, unpaired t-test, and Chi-square test were used to test for significant differences. Angiotensin converting enzyme inhibitor (ACE-I), Acetylsalicylic acid (ASA), angiotensin receptor blocker (ARB), coronary artery disease (CAD), body mass index (BMI), mild therapeutic hypothermia (MTH)*.

**Table 2:** Routine blood testing at pPCI.

**Parameter, [normal range]**	**Normothermia (*n* = 54)**	**MTH (*n* = 47)**
White blood cell count, G/l, [4–10]	11.9 ± 3.7	11.5 ± 3.7
CRP, nmol/l, [<48]	75.2 ± 125.7	114.3 ± 386.7
Cholesterol, mmol/l, [<5.1]	4.7 ± 1	5 ± 0.9
LDL, mmol/l, [<4.1]	2.8 ± 1	3 ± 0.7
HDL, mmol/l, [>1.4]	1 ± 0.3	1.1 ± 0.3
Triglycerides, mmol/l, [<1.6]	1.7 ± 1.1	1.7 ± 0.9
Creatinine, μmol/l, [44–80]	81.7 ± 20.8	79.9 ± 28.2
Creatine kinase, U/l, [<190]	273.9 ± 390.2	275.3 ± 633.1
Creatine kinase MB fraction, U/l, [<24]	73.8 ± 57.2	68.9 ± 87
Troponin T, μg/l, [0–0.014]	0.22 ± 0.57	0.44 ± 1.79
Fibrinogen, mg/dl, [200–400]	381.2 ± 94.6	374.9 ± 109
Lactate, mmol/l, [0.5–2.2]	2.1 ± 1.5	2 ± 1.2

*Data are provided as mean ± standard deviation. Reference values are given in parentheses. Mann-Whitney-U-test and unpaired t-test were used to test for significant differences. CRP, C-reactive protein; HDL, high density lipoprotein; LDL, low density lipoprotein; MTH, mild therapeutic hypothermia; pPCI, primary percutaneous coronary intervention*.

### Plasmatic Coagulation, Platelet Count, and Mean Platelet Volume

**Figure 1:**
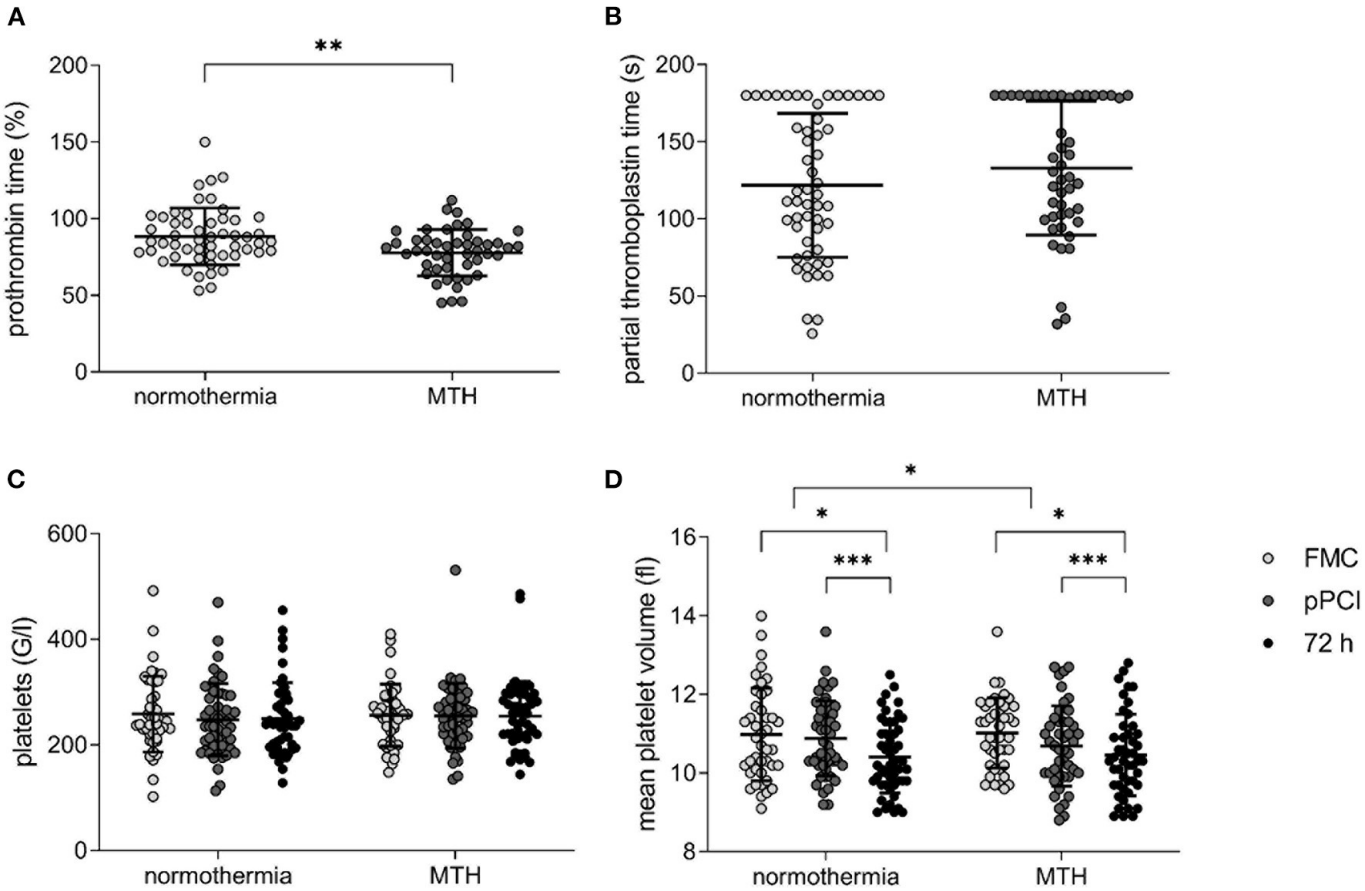
Platelet count, mean platelet volume and bleeding times in normothermia and MTH patients. **(A)** prothrombin time in percent (normothermia *n* = 53, MTH *n* = 47) and **(B)** partial thromboplastin time in seconds (normothermia *n* = 53, MTH *n* = 47) measured at pPCI. **(C)** platelet count in G/l (equals 1,000/ml; normothermia *n* = 51, MTH *n* = 45) and **(D)** MPV in fl (normothermia *n* = 51, MTH *n* = 45) from FMC until 72 h after pPCI. For **(A)**, unpaired *t*-test was used. For **(B)**, Mann-Whitney-*U*-test was used. For **(C,D)**, ANOVA with repeated measures was used. First medical contact (FMC), mild therapeutic hypothermia (MTH), mean platelet volume (MPV), primary percutaneous coronary intervention (pPCI). *p < 0.05, **p < 0.01, ***p < 0.001.

Patients in the MTH group displayed lower prothrombin time measured at pPCI, indicating a longer clotting time under MTH (normothermia: 88.42 ± 18.42%, MTH: 77.83 ± 15.15%; *p* < 0.01; Figure 1A). No significant differences were detected regarding partial thromboplastin time (normothermia: 121.86 ± 46.61 s, MTH: 132.98 ± 43.46 s; not significant; Figure 1B).

Platelet count was not different between the groups and remained unchanged over time from FMC until 72 h after pPCI (Figure 1C). Mean platelet volume declined in both groups from FMC to 72 h after pPCI. This decline in volume was greater in the MTH group (Δ normothermia: −0.12 ± 1.5 fl, Δ MTH: −0.28 ± 1.53 fl; *p* < 0.05; Figure 1D).

### Platelet Reactivity of MTH Patients Is Increased *ex vivo* Compared to Normothermia

**Figure 2:**
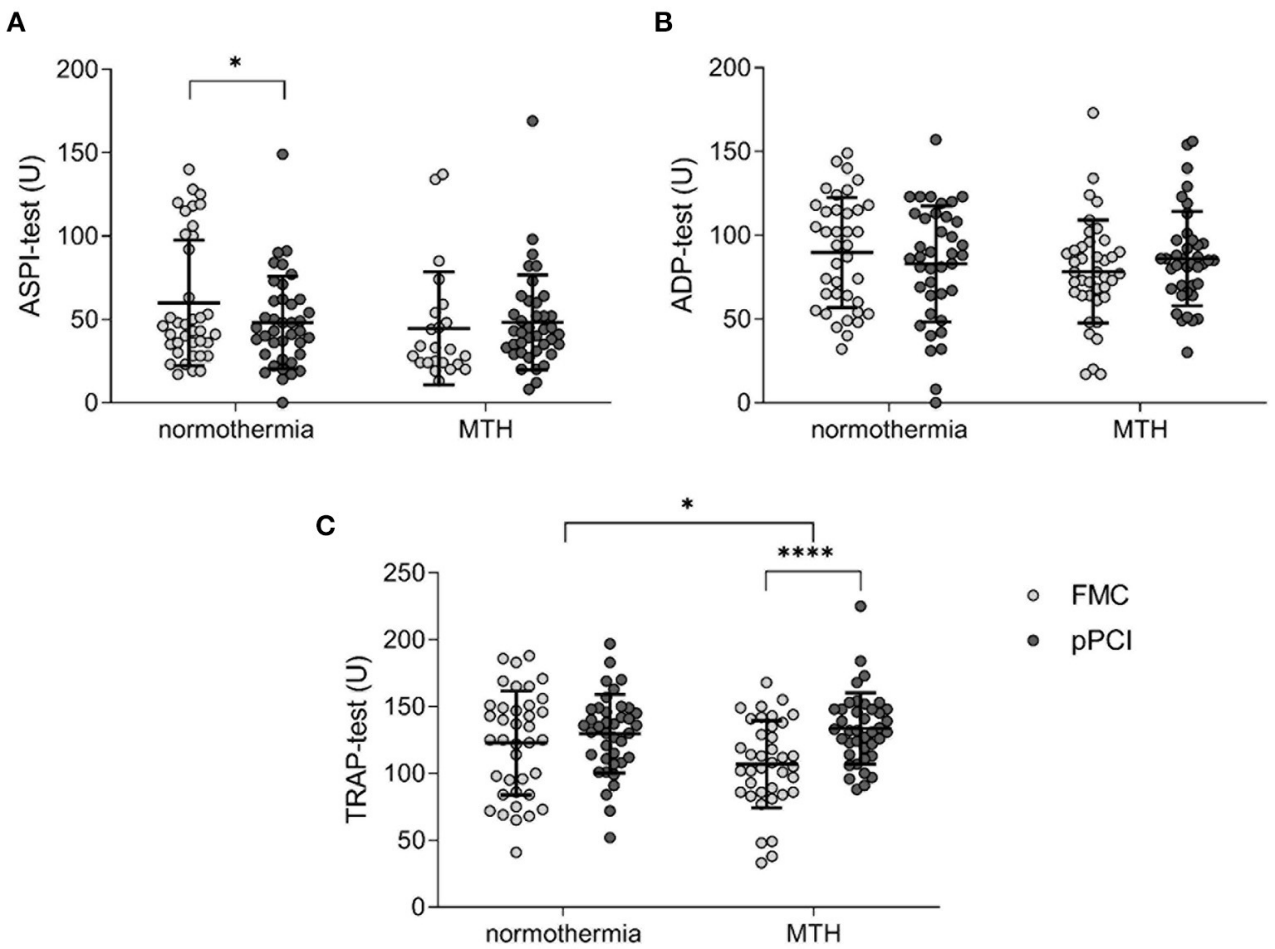
Platelet aggregometry. **(A)** ASPI-test (normothermia *n* = 38, MTH *n* = 35), **(B)** ADP-test (normothermia *n* = 38, MTH *n* = 35), and **(C)** TRAP-test (normothermia *n* = 38, MTH *n* = 34) were performed with whole blood obtained at first medical contact and pPCI. Results are illustrated as area under the curve units. For **(A)**, Wilcoxon signed rank test was used. For **(B,C)**, paired and unpaired *t*-tests were used. First medical contact (FMC), mild therapeutic hypothermia (MTH), primary percutaneous coronary intervention (pPCI), units (U). *p < 0.05, ****p < 0.0001.

Inhibition of (1) cyclooxygenase 1 (ASPI-test), (2) ADP receptor P2Y12 (ADP-test) and (3) and thrombin receptor (TRAP-test) were determined in platelets from FMC and pPCI blood samples. While cyclooxygenase 1 was significantly inhibited between FMC and pPCI in the normothermia STEMI cohort (FMC 59.89 ± 37.66 U vs. pPCI 47.39 ± 27.69 U; *p* < 0.05; Figure 2A), no inhibition was observed in the MTH cohort. TRAP-induced platelet reactivity increased significantly from FMC to pPCI in the MTH group (FMC 106.89 ± 32.67 U vs. pPCI 133.58 ± 27.18 U; *p* < 0.0001; Figure 2C), but remained unchanged in the normothermia group, resulting in a significant difference between the groups (Δ normothermia: 6.5 ± 37.21 U, Δ MTH: 26.68 ± 32.31 U; *p* < 0.05; Figure 2C). ADP-mediated platelet activation behaved in the same way (decrease in the normothermia group and increase in the MTH group at FMC and pPCI) but differences were not significant (Figure 2B).

### ADP Levels Are Increased in STEMI Patients Treated With MTH

**Figure 3:**
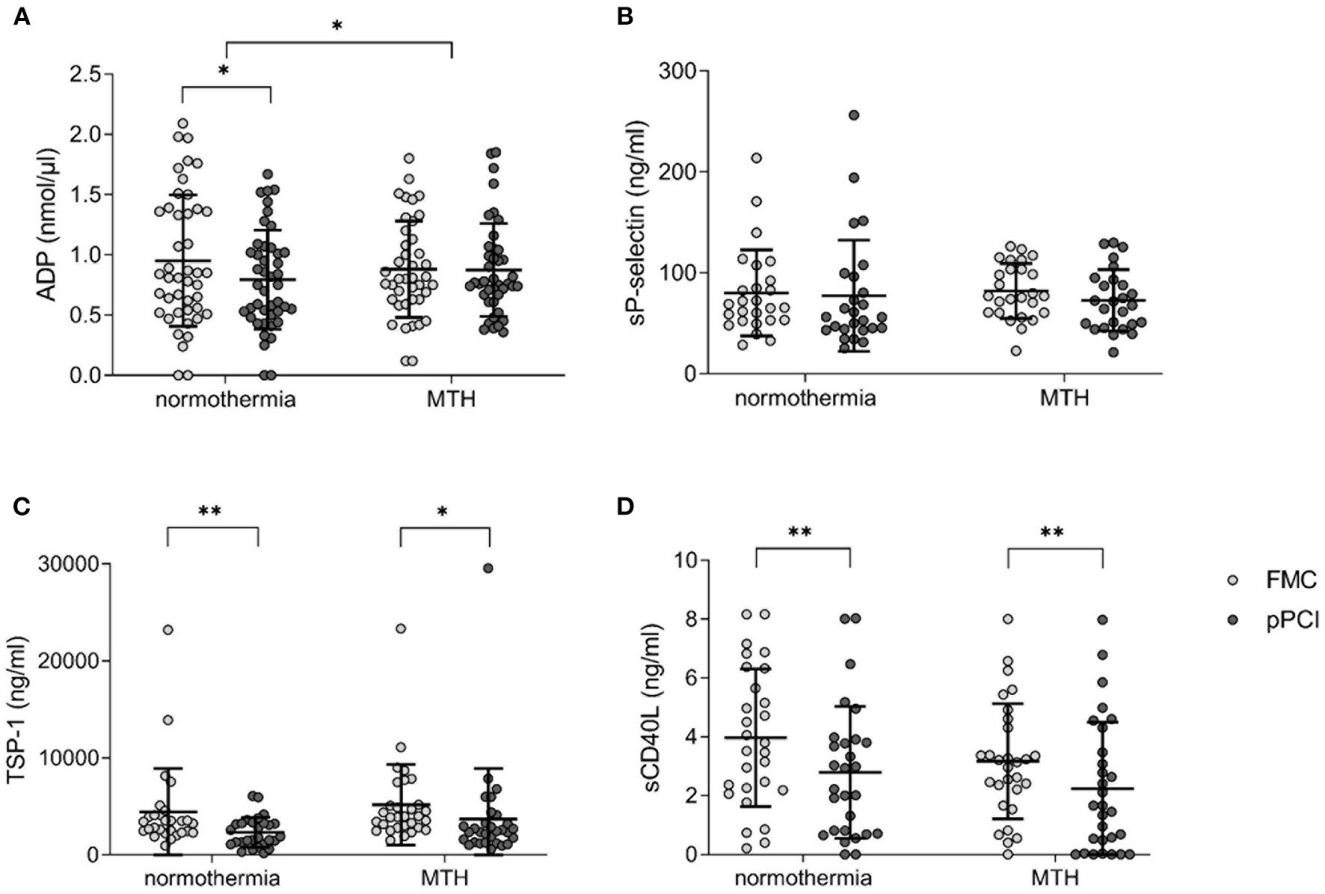
Soluble platelet activation markers. **(A)** ADP concentration given in nmol/μl (normothermia *n* = 44, MTH *n* = 41). **(B)** sP-selectin (normothermia *n* = 26, MTH *n* = 26), **(C)** TSP-1 (normothermia *n* = 28, MTH *n* = 30), and **(D)** sCD40L (normothermia *n* = 28, MTH *n* = 29) concentrations are presented as ng/ml from FMC and pPCI plasma. For **(A)**, paired and unpaired *t*-tests were used. For **(B–D)**, Wilcoxon signed rank tests were used. Adenosine diphosphate (ADP), first medical contact (FMC), mild therapeutic hypothermia (MTH), primary percutaneous coronary intervention (pPCI), soluble CD40 ligand (sCD40L), soluble P-selectin (sP-selectin), thrombospondin-1 (TSP-1). *p < 0.05, **p < 0.01.

We measured ADP levels, sP-selectin, TSP-1 and sCD40L at FMC and pPCI by ELISA. Normothermia patients displayed decreased levels of ADP at pPCI compared to FMC (FMC 0.94 ± 0.56 nmol/μl vs. pPCI 0.78 ± 0.43 nmol/μl; *p* < 0.05; Figure 3A), while MTH patients remained unchanged, with a significant intergroup delta difference in ADP levels from FMC to pPCI (Δ normothermia: −0.16 ± 0.46 nmol/μl, Δ MTH: −0.01 ± 0.4 nmol/μl; *p* < 0.05; Figure 3A). Soluble P-selectin, TSP-1, sCD40L did not differ between patients treated with MTH and normothermia patients (Figures 3B–D). However, TSP-1 (normothermia: FMC 4,450.99 ± 4,466.21 ng/ml vs. pPCI 2,343.75 ± 1,554.46 ng/ml; *p* < 0.01; MTH: FMC 5,185.68 ± 4,154.88 ng/ml vs. pPCI 3,701.96 ± 5,208.03 ng/ml; *p* < 0.05; Figure 3C) and sCD40L (normothermia: FMC 3.97 ± 2.34 ng/ml vs. pPCI 2.79 ± 2.25 ng/ml; *p* < 0.01; MTH: FMC 3.17 ± 1.95 ng/ml vs. pPCI 2.24 ± 2.25 ng/ml; *p* < 0.01; Figure 3D) plasma levels decreased in both groups from FMC to pPCI. It has been shown that angiotensin receptor blockade and statins reduced sCD40L in patients (21) and p-selectin levels in animals (22). We did not detect any differences in soluble platetet activation marker levels between patients with and without such preexisting medication (data not shown).

### MTH Influences PAC-1 and P-Selectin Platelet Receptor Expression

**Figure 4:**
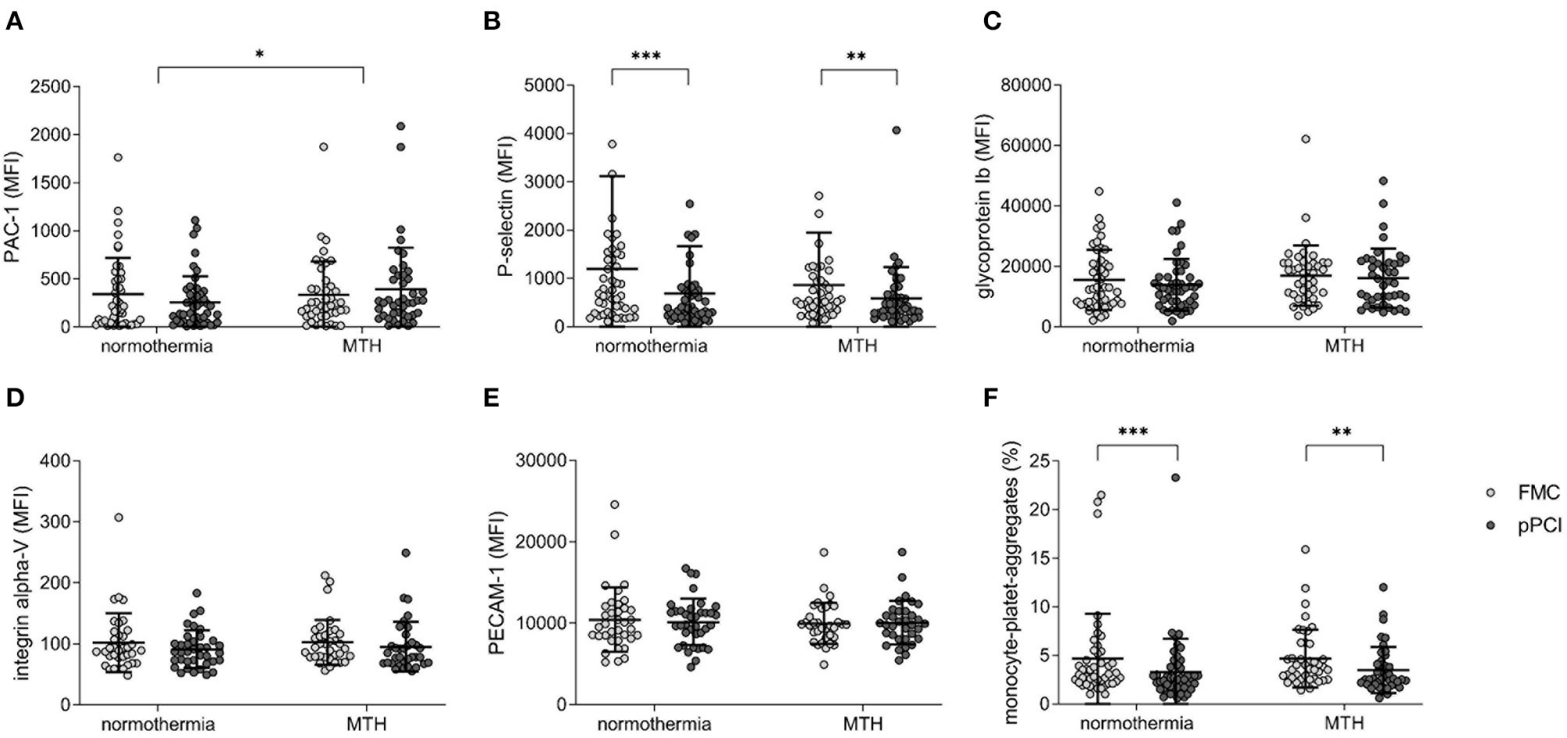
Platelet receptor expression. **(A)** Glycoprotein IIb/IIIa (PAC-1; normothermia *n* = 46, MTH *n* = 43), **(B)** P-selectin (CD62p; normothermia *n* = 46, MTH *n* = 43), **(C)** Glycoprotein Ib (CD42b; normothermia *n* = 46, MTH *n* = 43), **(D)** Integrin α-V (CD51; normothermia *n* = 36, MTH *n* = 35), **(E)** PECAM-1 (CD31; normothermia *n* = 36, MTH *n* = 34) expression on platelets at FMC and pPCI comparing normothermia and MTH patients. Data are expressed as MFI. **(F)** MPA (normothermia *n* = 40, MTH *n* = 42) in the normothermia and MTH group in percentage of total platelet counts. For **(A–F)**, Wilcoxon signed rank tests and Mann-Whitney-*U*-tests were used. First medical contact (FMC), mean fluorescence intensity (MFI), monocyte-platelet-aggregates (MPA) mild therapeutic hypothermia (MTH), procaspase activating compound-1 (PAC-1), platelet endothelial cell adhesion molecule-1 (PECAM-1), primary percutaneous coronary intervention (pPCI). *p < 0.05, **p < 0.01, ***p < 0.001.

Platelet expression of PAC-1 and P-selectin at FMC and at pPCI were assessed by flow cytometry. PAC-1 expression decreased from FMC to pPCI in normothermia patients while it increased in MTH patients (Δ normothermia: −81.65 ± 381.25 MFI (mean fluorescence intensity), Δ MTH: 57.56 ± 471.99 MFI; *p* < 0.05; Figure 4A).

No group difference was detected for P-selectin (Figure 4B), glycoprotein Ib (Figure 4C), integrin alpha-V (Figure 4D), PECAM-1 (Figure 4E), and MPA (Figure 4F). P-selectin expression was decreased in both groups at FMC and pPCI (normothermia: FMC 1,192.59 ± 1,924.01 MFI vs. pPCI 696.17 ± 989.27 MFI; *p* < 0.001; MTH: FMC 858.60 ± 1,092.52 MFI vs. pPCI 586.58 ± 647.66 MFI; *p* < 0.01; Figure 4B). In both groups, MPA decreased from FMC to pPCI (normothermia: FMC 4.65 ± 4.66 % vs. pPCI 3.25 ± 3.49 %; *p* < 0.001; MTH: FMC 4.66 ± 2.99 % vs. pPCI 3.50 ± 2.38 %; *p* < 0.01; Figure 4F).

### MTH Is Associated With a Lower pH Value and Longer Clotting Times

**Figure 5:**
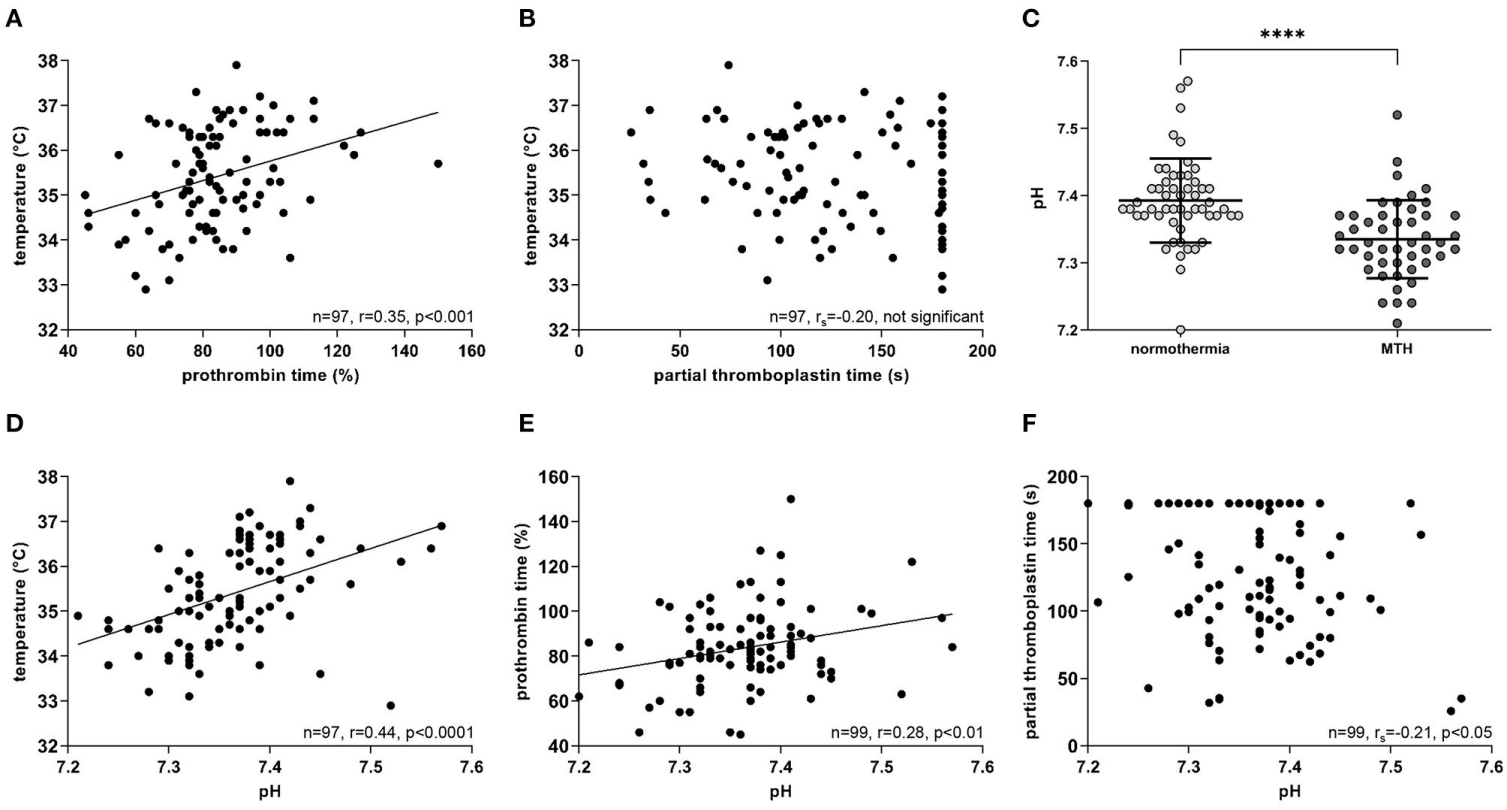
Correlations of temperature, bleeding time and pH. All measurements were at the time of reperfusion during pPCI. **(A)** Temperature correlated positively with prothrombin time (*n* = 97), but not with, **(B)** partial thromboplastin time (*n* = 97). **(C)** MTH patients had significantly lower pH values compared to the normothermia group (normothermia *n* = 53, MTH *n* = 47). **(D)** pH correlated positively with temperature (*n* = 97) and **(E)** prothrombin time (n=99), whereas negatively with, **(F)** partial thromboplastin time (*n* = 99). For **(A,D,E)**, Pearson correlation was used. For **(B,F)** Spearman rank correlation was used. For **(C)**, Mann-Whitney-*U*-test was used. Celsius (°C), mild therapeutic hypothermia (MTH), primary percutaneous coronary intervention (pPCI), seconds (s). ****p < 0.0001.

Body temperature at reperfusion correlated positively with prothrombin time (*n* = 97; *r* = 0.35; *p* < 0.001; Figure 5A). The same trend was observed for partial thromboplastin time, but was not significant (*n* = 97; *r*_*s*_ = −0.20; not significant; Figure 5B).

Blood pH measured at pPCI was significantly lower in patients treated with MTH than in normothermia patients (normothermia: 7.39 ± 0.06 mmol/l, MTH: 7.33 ± 0.06 mmol/l; *p* < 0.0001; Figure 5C). Lower blood pH correlated significantly with lower patient temperatures (*n* = 97; *r* = 0.44; *p* < 0.0001; Figure 5D). Furthermore, pH correlated positively with prothrombin time (*n* = 99; *r* = 0.28; *p* < 0.01; Figure 5E) and on the contrary, negatively with partial thromboplastin time (*n* = 99; *r*_*s*_ = −0.21; *p* < 0.05; Figure 5F).

## Discussion

In the present study, we sought to investigate platelet function and plasma coagulation of STEMI patients in response to MTH in a controlled, prospective trial. We are demonstrating opposing effects of MTH on primary and secondary hemostasis—higher platelet reactivity and longer clotting times in cooled patients. The observation of attenuated platelet inhibition by dual antiplatelet therapy under conditions of MTH is important because effective platelet inhibition is an essential treatment step prior to implantation of stents.

MTH was hoped to reduce infarct size in STEMI. Up to now, none of the published trials except for a small feasibility trial (8) could prove a significant reduction of infarct size (6, 13). Patients presenting early after symptom onset with a large myocardium at risk who reach a target temperature below 35°C early may benefit most from MTH (12). Therefore, MTH was initiated at FMC in the STATIM trial, and 80% of the patients reached the target temperature at reperfusion. However, no difference in CMR-measured outcome variables could be observed (13).

The effect of MTH on hemostasis and the occurrence of hemorrhage is controversial. Several studies suggested a prolonged clot initiation during hypothermia in cardiac arrest patients (16, 23). Two meta-analyses did not detect a higher risk for bleeding due to MTH, neither in cardiac arrest (24), nor in STEMI (12). In the STATIM trial, we also did not observe more bleedings in patients treated with MTH compared to normothermia patients.

Hemostasis is a complex process, involving platelets (primary hemostasis) and fibrin aggregation (secondary hemostasis). In the STATIM trial, secondary hemostasis was evaluated by prothrombin time and partial thromboplastin time. We observed a significantly longer prothrombin time in the MTH group compared to normothermia patients (Figure 1A). Partial thromboplastin time was prolonged in both groups at pPCI due to heparinization, but was not significantly longer in patients who underwent MTH (Figure 1B). Other studies did not observe significant changes in coagulation during MTH (15, 25).

Platelets play a crucial role in the coagulation system and thrombus formation. Growing evidence indicates increased platelet activity under MTH (26, 27). Cooling of platelets *in vitro* below 15°C resulted in a volume increase and formation of pseudopods (28). Lower body temperature of 32–33°C lead to a decrease in platelet count, possibly caused by hepatic and splenic sequestration (16, 29). Platelet counts of patients in the STATIM trial remained unchanged (Figure 1C). Shape change and granule release are indirectly monitored by measuring MPV. We detected smaller platelets from FMC onwards to pPCI and 72 h in both groups. The drop in platelet size was more pronounced in patients of the MTH group (Figure 1D). *In vitro*, increased platelet aggregation between 30 and 34°C was shown (26). In *ex vivo* platelet aggregometry, we found that platelet reactivity decreased from FMC to pPCI in the normothermia group, while it remained unchanged in the MTH group (Figure 2).

Furthermore, we measured the expression of platelet receptors at FMC and pPCI. PAC-1 (glycoprotein IIb/IIIa complex) is a crucial receptor for platelet aggregation. While PAC-1 expression decreased in normothermia patients until pPCI, it increased in MTH patients (Figure 4A). Consistently, increased platelet PAC-1 expression was observed in an *in vitro* study under hypothermia (30). P-selectin and MPA decreased significantly from FMC to pPCI in both treatment groups (Figures 4B,F). ADP reactivity in aggregometry correlated with platelet PAC-1 and P-selectin receptor expression (Supplementary Figure 1).

STEMI patients receive potent platelet-inhibitory medication early on. In the STATIM trial, acetylsalicylic acid and ADP inhibitors were administered at FMC. Therefore, reduced platelet reactivity is expected. However, it is known that effective platelet receptor inhibition is significantly delayed in STEMI after loading with dual anti-platelet medication (31, 32).

We observed mild reduction of platelet reactivity by aggregometry (Figure 2A) and decreasing PAC-1 expression in the normothermia STEMI patient cohort (Figure 4A). In contrast, MTH partially abrogated platelet inhibition (Figure 2C). This might be one adverse effect of MTH treatment which could explain its failure to improve functional outcome after STEMI. However, no stent thrombosis were observed in the STATIM trial.

ADP was identified as key mediator of hypothermia-induced platelet activation. *In vitro* experiments with hypothermia showed reduced ADP hydrolysis via decreased E-NTPDase1 activity, which resulted in higher plasmatic ADP levels and platelet activation (33). This is supported by our *in vivo* data because we observed significantly higher ADP plasma levels in the MTH than in the normothermia cohort (Figure 3A).

Our data demonstrate a significant correlation of lower temperature and lower prothrombin time (Figure 5A). Moreover, lower patient temperatures at reperfusion are associated with lower pH values (Figure 5D). Longer prothrombin time and partial thromboplastin time correlated significantly with lower pH (Figures 5E,F). Additionally, patients in the hypothermia group had significantly lower blood pH (Figure 5C). The decline of pH under hypothermia is considered to be a result of hyperlactataemia (34, 35), which is caused by increased fat metabolism (36). In an *in vitro* study with whole blood, acidosis impaired clotting times primarily under hypothermia, as it has a substantial effect on thrombin-generating kinetics (37). Our data from STEMI patients are in agreement with these data. We propose that MTH and subsequent changes in pH might impair secondary hemostasis in STEMI patients. However, further studies to clarify the detailed mechanism are warranted.

We did not observe an effect of our findings on hemostasis and bleeding events, which were overall low (Table 1).

In the STATIM trial, CMR was performed 2 ± 4 days after pPCI to measure functional outcome and infarct size (13). None of the platelet activation and plasmatic coagulation measurements correlated with CMR-measured variables.

In conclusion, we found that platelet reactivity was increased in patients treated with MTH, whereby non-degraded ADP in plasma might be an important mediator of platelet activation under hypothermia. Concurrently, secondary hemostasis was slightly impaired under MTH.

## Limitations

Temperature changes, while transporting or preparing patient samples could have alleviated group differences. Yet, all samples were processed immediately and no additional cooling or heating steps were applied. Only patients treated with MTH received ice-cold saline, buspirone, and meperidine, which might have an impact on the observed effects. All data from this prospective human STEMI study are observational and do not suffice to draw conclusions on the mechanism of MTH-induced changes in primary and secondary hemostasis.

## Data Availability Statement

The original contributions presented in the study are included in the article/Supplementary Material, further inquiries can be directed to the corresponding author/s.

## Ethics Statement

The studies involving human participants were reviewed and approved by Ethics Committee of the Medical University of Vienna, Austria (approval number: 1497/2012). The patients/participants provided their written informed consent to participate in this study.

## Author Contributions

TS, FS, CT, IL, and AM designed the study and developed the study concept. FS and CT were responsible for patient inclusion in the clinical trial. TS, TH, DS, CT, AM, and IL executed the acquisition of samples. TS, TH, AO and DS were involved in the laboratory processing and methods. TS, TH, AO and AM processed and analyzed the data. TS wrote the original draft and designed all figures and tables. IL and AM reviewed and edited the manuscript. IL and AM supervised TS in the execution of the study and were responsible for acquisition of the funding. All authors contributed to the article and approved the submitted version.

## Conflict of Interest

The authors declare that the research was conducted in the absence of any commercial or financial relationships that could be construed as a potential conflict of interest.

## Abbreviations

ACE-I: Angiotensin converting enzyme inhibitor
ACS: Acute coronary syndrome
ADP: Adenosine diphosphate
ARB: Angiotensin receptor blocker
ASA: Acetylsalicylic acid
BMI: Body mass index
CMR: Cardiac magnetic resonance
EDTA: Ethylenediaminetetraacetic acid
ELISA: Enzyme-linked immunosorbent assay
FMC: First medical contact
MFI: Mean fluorescence intensity
MPA: Monocyte-platelet-aggregates
MTH: Mild therapeutic hypothermia
NSAID: Non-steroidal anti-inflammatory drugs
PAC-1: Procaspase activating compound-1
PECAM-1: Platelet endothelial cell adhesion molecule-1
pPCI: Primary percutaneous coronary intervention
sCD40L: Soluble CD40 ligand
sP-selectin: Soluble P-selectin
SD: Standard deviation
STEMI: ST-segment elevation myocardial infarction
TIMI: Thrombolysis in myocardial infarction grade flow
TRAP: Thrombin receptor activating peptide-6
TSP-1: Thrombospondin 1.
