# Supplementary material for: Mild Therapeutic Hypothermia Alters Hemostasis in ST Elevation Myocardial Infarction Patients
Source: Front Cardiovasc Med. 2021 Jul 6;8:707367. doi: 10.3389/fcvm.2021.707367 (PMC8290912; doi:10.3389/fcvm.2021.707367)
Supplement: Supplementary file 1 [file Data_Sheet_1.PDF]

Supplementary Material

1.1 Supplementary Figures

Supplemental Figure 1. Correlation of ADP reactivity with platelet PAC-1 and P-selectin receptor expression. (A), ADP reactivity in units correlated positively with platelet receptor expression of PAC-1 given as MFI (n=77) as well as with, (B), platelet P-selectin receptor expression in MFI (n=77). All depicted values were measured at the time of reperfusion during pPCI. Mean fluorescence intensity (MFI), primary percutaneous coronary intervention (pPCI), units (U).
